# A Case of Vancomycin-Induced Immune Thrombocytopenia

**DOI:** 10.7759/cureus.7940

**Published:** 2020-05-03

**Authors:** Kira N MacDougall, Sara Parylo, Alisa Sokoloff

**Affiliations:** 1 Internal Medicine, Staten Island University Hospital, Northwell Health, New York, USA; 2 Hematology/Oncology, Staten Island University Hospital, New York, USA; 3 Hematology/Oncology, Staten Island University Hospital, Northwell Health, New York, USA

**Keywords:** immune thrombocytopenia, vancomycin, vancomycin-induced immune thrombocytopenia, drug-induced immune thrombocytopenia

## Abstract

Vancomycin-induced immune thrombocytopenia (ITP) is a rare, potentially life-threatening complication from an antibiotic frequently used in medical practice. We report a case of an 81-year-old male with recent removal of an infected right knee prosthesis and insertion of an articulating antibiotic spacer, presenting from rehabilitation for severe thrombocytopenia (1 X 10^3^/µL). The patient’s thrombocytopenia was initially falsely attributed to rifampin-induced ITP, a much more common cause of drug-induced thrombocytopenia. Only later, after a second precipitous drop in platelet count, vancomycin was correctly identified as the culprit. The patient’s serum was tested for drug-dependent platelet antibodies with and without vancomycin. A positive reaction for IgG was detected by flow cytometry in the absence of vancomycin, which was potentiated in the presence of vancomycin. The result indicated the presence of vancomycin-dependent and nondrug-dependent platelet reactive antibodies and confirmed the diagnosis of vancomycin-induced ITP. In this case, the correct diagnosis was masked by the simultaneous administration of two drugs that cause drug-induced ITP and highlights the importance of early recognition of rare, vancomycin-induced ITP.

## Introduction

In the hospitalized patient, acquired thrombocytopenia is a relatively common clinical phenomenon that poses a unique diagnostic challenge due to the broad differential diagnosis. One such etiology is drug-induced thrombocytopenia, which can occur by several mechanisms including direct bone marrow suppression, organ toxicity, or drug-induced immune thrombocytopenia (DITP). DITP is caused by drug-dependent platelet antibodies that cause accelerated platelet destruction by the reticuloendothelial system, often resulting in severe thrombocytopenia, and in some cases, life-threatening bleeding [1].

The drug classes most commonly implicated in DITP are quinines, sulfonamides, nonsteroidal anti-inflammatory drugs, anticonvulsants, disease-modifying antirheumatic drugs, and diuretics [2]. There are only a few rare cases in the literature of vancomycin being implicated as a cause of antibody-mediated thrombocytopenia [3-8]. Prior to a pivotal study conducted by Von Drygalski et al., there was only limited evidence that the mechanism was immune mediated [9]. We are now able to detect drug-dependent antiplatelet antibodies by flow cytometry and diagnose vancomycin-induced immune thrombocytopenia (ITP). Due to the frequency of vancomycin use, it is important to expand our knowledge on the subject and educate ourselves regarding the management of this potentially life-threatening condition.

## Case presentation

An 81-year-old male was transferred to the medical service from inpatient physical rehabilitation after routine blood work revealed severe thrombocytopenia. He was undergoing rehabilitation following removal of an infected right knee prosthesis and insertion of an articulating antibiotic spacer. His medical history includes hypertension, dyslipidemia, and a chronic right popliteal deep vein thrombosis. Following the surgical intervention, the patient was discharged to the inpatient physical rehabilitation floor and started on cefepime 2,000 mg intravenously every eight hours, vancomycin 1,500 mg intravenously every 12 hours, and rifampin 300 mg intravenously every 12 hours.

**Figure 1:**
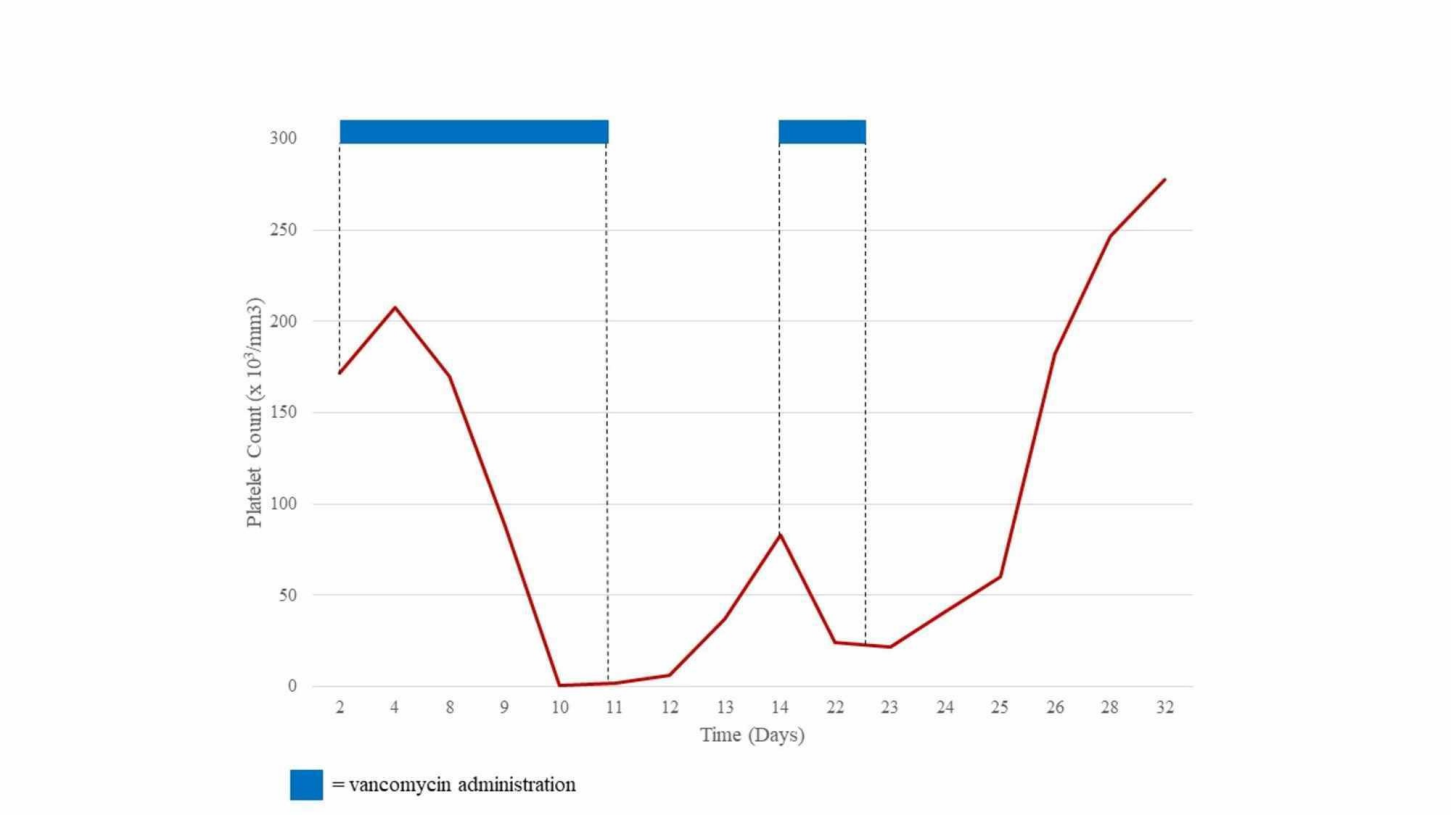
Platelet Count in Relation to Vancomycin Administration

Prior to the initiation of antibiotic therapy, platelet count was 172 X 10^3^/µL (Table 1). Routine lab work done in the rehabilitation unit showed a precipitous drop in platelets from 170 X 10^3^/µL on hospital day 8 to 88 X 10^3^/µL on hospital day 9, and then to 1 X 10^3^/µL on hospital day 10 (Figure 1).Repeat blood work confirmed a platelet count of 1 X 10^3^/µL. At no point did the patient receive any heparin-based products. The hematology consultants reviewed the peripheral smear which demonstrated very few platelets and no schistocytes. The patient denied any bleeding events, hemoptysis, hematemesis, melena, or hematochezia. To the patient’s knowledge, he had never had thrombocytopenia before. Physical exam revealed minor petechia on his right lower extremity.

**Table 1:** Laboratory Data on Admission WBC = white blood cell; RBC = red blood cell; Hb = hemoglobin; Hct = hematocrit.

WBC	12.36	K/µL	Sodium	142	mEq/L
Neutrophils	62.4	%	Potassium	4.1	mEq/L
Lymphocytes	21.3	%	Chloride	105	mEq/L
Monocytes	13.8	%	Blood urea nitrogen	10	mg/dL
Eosinophils	1.8	%	Creatinine	0.8	mg/dL
Basophils	0.3	%	Glucose	116	mg/dL
RBC	3.48	M/µL	Calcium	8.6	mg/dL
Hb	10.6	g/dL			
Hct	31.9	%			
Platelets	172	K/µL			

Following transfusion with one unit of platelets, repeat complete blood count revealed a platelet count of 2 X 10^3^/µL the following day. Lack of improvement following transfusion suggested ITP. The patient was then started on prednisone at 1 mg/kg and intravenous immunoglobulin (IVIG) at 0.4g/kg over the next four days. The patient’s medications were reviewed and rifampin, well known for causing ITP, was immediately discontinued. Our infectious disease consultants recommended switching to daptomycin 8 mg/kg intravenously every 24 hours. At this time, cefepime and vancomycin were also discontinued. The platelet count recovered over the next five days, and the patient’s thrombocytopenia was incorrectly attributed to rifampin-induced ITP.

Antibiotic therapy was again changed in anticipation for the patient’s discharge home. On the final day of hospitalization, the patient’s platelets improved to 88 X 10^3^/µL. Daptomycin was discontinued, and the patient was re-started on vancomycin 1,250 mg intravenously every 12 hours to complete a six-week course and prednisone 90 mg daily. The following day, outpatient blood work revealed a platelet count of 24 X 10^3^/µL and the patient was re-admitted for refractory ITP with plans to initiate rituximab 375 mg/m^2^ weekly x 4 doses. At this point, review of the literature revealed rare case reports of vancomycin-induced ITP and vancomycin was considered as the causative agent and was discontinued. In the following days, platelets subsequently rebounded to 41 X 10^3^/µL, 60 X 10^3^/µL, 182 X 10^3^/µL, and 278 X 10^3^/µL. The patient had no clinically significant bleeding episodes during these events. The patient’s thrombocytopenia resolved, and the patient was discharged on doxycycline 100 mg intravenously every 12 hours with a presumptive diagnosis of vancomycin-induced ITP.

Vancomycin-induced platelet antibody testing was sent to an outside laboratory. The patient’s serum was tested for drug-dependent platelet antibodies with and without vancomycin. A positive reaction for IgG was detected by flow cytometry in the absence of vancomycin which was potentiated in the presence of vancomycin. These results indicate the presence of vancomycin-dependent and nondrug-dependent platelet reactive antibodies and confirm the diagnosis of vancomycin-induced ITP.

## Discussion

We present the case of an 81-year-old male being treated with a combination of cefepime, rifampin, and vancomycin following removal of an infected right knee prosthesis and insertion of an articulating antibiotic spacer. He was found to have severe thrombocytopenia on routine blood work, which improved after discontinuation of rifampin and vancomycin. The medical team initially suspected rifampin-induced ITP. However, upon re-administration of vancomycin, platelet count again plummeted. Due to the rarity of vancomycin-induced ITP, it was not initially included in the differential diagnosis. Only after a second drop in platelet count was this unlikely cause of thrombocytopenia suspected. The diagnosis was later confirmed by the detection of vancomycin-dependent antiplatelet antibody by flow cytometry.

This case is unique for several reasons. First, the simultaneous administration of two drugs that cause DITP complicated the clinical picture. Second, the absence of clinically significant bleeding is unusual compared to many previous cases of DITP described in the literature. Finally, in addition to the vancomycin-dependent antiplatelet antibody detected by flow cytometry, we have documented a clear temporal relationship of platelet decline upon exposure and re-exposure to vancomycin.

DITP is a unique clinical syndrome that is caused by drug-dependent platelet antibodies that promote clearance by the reticuloendothelial system. Epidemiologic studies suggest that approximately 10 persons per million are affected by DITP [2]. Historically, quinine and its isomer quinidine have been associated with drug-induced thrombocytopenia [10]. However, with advances in technology and the detection of drug-dependent antiplatelet antibodies by flow cytometry, more drugs are being identified.

DITP should be considered in any patient who presents with severe, unexplained thrombocytopenia. A thorough history, including all present and past drug exposures, is essential in establishing this diagnosis. DITP occurs five to ten days after initiation of a new drug, or within hours after re-exposure [1]. Severe thrombocytopenia with a platelet count less than 20 X 10^3^/µL is typically observed [1]. Positive laboratory testing demonstrating drug-dependent antiplatelet antibodies confirms the diagnosis. After discontinuing the offending drug, thrombocytopenia usually begins to recover within one to two days, with complete recovery after one week [11]. In patient with severe thrombocytopenia and bleeding, other supportive measures including high-dose IVIG and corticosteroids may be indicated. Most importantly, in patients with confirmed DITP, patients must be counseled to avoid the drug indefinitely, as drug-dependent antibodies may persist in the blood for years [11]. 

## Conclusions

This case implicates vancomycin-dependent antiplatelet antibodies as the cause of the patient’s severe thrombocytopenia. Vancomycin-induced ITP is rare and can easily be overlooked. Given the frequency of vancomycin use in today’s clinical practice, increased awareness of this potentially life-threatening condition is warranted.
